# The AFHSC-Division of GEIS Operations Predictive Surveillance Program: a multidisciplinary approach for the early detection and response to disease outbreaks

**DOI:** 10.1186/1471-2458-11-S2-S10

**Published:** 2011-03-04

**Authors:** Clara J Witt, Allen L Richards, Penny M Masuoka, Desmond H Foley, Anna L Buczak, Lillian A Musila, Jason H Richardson, Michelle G Colacicco-Mayhugh, Leopoldo M Rueda, Terry A Klein, Assaf Anyamba, Jennifer Small, Julie A Pavlin, Mark M Fukuda, Joel Gaydos, Kevin L Russell, the AFHSC-GEIS Predictive Surveillance Writing Group

**Affiliations:** 1 Armed Forces Health Surveillance Center, 503 Robert Grant Avenue, Silver Spring, MD 20910, USA; 2 Department of Rickettsial Diseases Research Program, Naval Medical Research Center, 503 Robert Grant Avenue, Silver Spring, MD 20910, USA; 3 Department of Preventive Medicine and Biometrics, Uniformed Services University of the Health Sciences, 4301 Jones Bridge Road, Bethesda, MD 20814, USA; 4 Division of Entomology, Walter Reed Biosystematics Unit, Walter Reed Army Institute of Research, 503 Robert Grant Avenue, Silver Spring, MD 20910, USA; 5 Johns Hopkins University Applied Physics Laboratory, 11100 Johns Hopkins Road, Laurel, MD 20723, USA; 6 Kenya Medical Research Institute Centre for Virus Research, Post Office Box 54628, Nairobi, Kenya; 7 U.S. Army Medical Research Unit-Kenya, Nairobi Unit 64109, APO AE 09831-4109, USA; 8 Armed Forces Research Institute of Medical Sciences, 315/6 Rajvithi Road, Bangkok, Thailand 10400; 9 Force Health Protection and Preventive Medicine, 65th Medical Brigade, Unit 15281, APO AP 96205-5281, USA (Republic of Korea; 10 NASA Goddard Space Flight Center, Biospheric Sciences Branch, GIMMS Group, Code 614.4, Greenbelt, MD 20771, USA; 11 Navy and Marine Corps Public Health Center, 620 John Paul Jones Circle, Suite 1100, Portsmouth, VA 23708-2103, USA; 12 U.S. Department of Agriculture-Center for Medical, Agricultural & Veterinary Entomology, 1600/1700 Southwest 23rd Drive, Gainesville, FL 32608, USA; 13 Naval Medical Research Unit Number 3, Extension of Ramses Street, Adjacent to Abbassia Fever Hospital, Postal Code 11517, Cairo, Egypt; 14 U.S. Army Medical Research Unit-Kenya, Kisumu Unit 8900, Department of Entomology and Vector-Borne Diseases, Box 6814, APO AE 09831, USA; 15 Naval Medical Research Unit Number 2, Komplek P2M/PLP – LITBANGKES JI. Percetakan Negara Number 29, Jakarta, 10560, Indonesia; 16 U.S. Naval Medical Research Center Detachment, Centro Medico Naval “CEMENA,” Av. Venezuela CDRA 36, Callo 2, Lima, Peru; 17 Division of Preventive Medicine, Walter Reed Army Institute of Research, 503 Robert Grant Avenue, Silver Spring, MD 20910, USA

## Abstract

The Armed Forces Health Surveillance Center, Division of Global Emerging Infections Surveillance and Response System Operations (AFHSC-GEIS) initiated a coordinated, multidisciplinary program to link data sets and information derived from eco-climatic remote sensing activities, ecologic niche modeling, arthropod vector, animal disease-host/reservoir, and human disease surveillance for febrile illnesses, into a predictive surveillance program that generates advisories and alerts on emerging infectious disease outbreaks. The program’s ultimate goal is pro-active public health practice through pre-event preparedness, prevention and control, and response decision-making and prioritization. This multidisciplinary program is rooted in over 10 years experience in predictive surveillance for Rift Valley fever outbreaks in Eastern Africa. The AFHSC-GEIS Rift Valley fever project is based on the identification and use of disease-emergence critical detection points as reliable signals for increased outbreak risk. The AFHSC-GEIS predictive surveillance program has formalized the Rift Valley fever project into a structured template for extending predictive surveillance capability to other Department of Defense (DoD)-priority vector- and water-borne, and zoonotic diseases and geographic areas. These include leishmaniasis, malaria, and Crimea-Congo and other viral hemorrhagic fevers in Central Asia and Africa, dengue fever in Asia and the Americas, Japanese encephalitis (JE) and chikungunya fever in Asia, and rickettsial and other tick-borne infections in the U.S., Africa and Asia.

## Background

The morbidity and mortality associated with infectious disease outbreaks, which are directly or indirectly linked to ecologic or climate events and trends, pose a growing problem for global public health [1-3]. Many factors are associated with this growth: disease vector-habitat expansion due to environmental degradation and climate variability; changes in animal and human population dynamics that increase the risk of human exposure to infective pathogens; and the insufficiency of public health infrastructure in resource-limited settings to support and sustain routine infectious disease surveillance, prevention and control activities. The goal of the AFHSC-GEIS predictive surveillance program is to provide DoD decision makers with advanced awareness on emerging infectious disease threats, and thereby promote timely, science-based disease outbreak prevention, preparedness, and control-and-response action. The proof of concept is best exemplified by the recurring outbreaks of Rift Valley fever in Africa. These outbreaks have not posed direct threats to DoD forces, but in Africa and the Middle East they do cause significant agricultural and trade disruptions in addition to human morbidity and mortality. The economic, social and political destabilization associated with Rift Valley fever outbreaks in vulnerable states like Yemen, Somalia and Zimbabwe cannot be understated. Geographic stabilization benefits not just those directly affected, but also those, like the US military, who may have to respond to a crisis. In addition, the accidental or intentional importation into the US of Rift Valley fever or other vector-borne human health or agricultural threat would be a disaster to the US economy. DOD involvement in any Federal response to such an outbreak would be costly. Keeping these threats under surveillance and contained outside the US, is in the direct interest of the US[4,5].

The economic and human health devastation of the 1997 Rift Valley fever outbreak in Eastern Africa was not repeated in the 2006-2007 outbreak. This was largely due to the implementation of early, targeted interventions by the affected countries and the international community, prompted by AFHSC-GEIS early alerts. The result was a more contained outbreak and a quicker return to regional economic, social and political stability.

**Figure 1:**
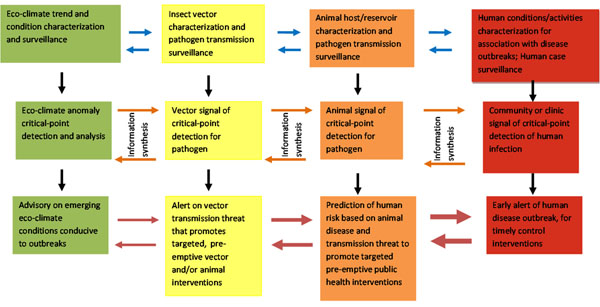
**AFHSC-GEIS Predictive Surveillance Program Boxes represent model components:** Green – eco-climate, yellow – vector, orange – animal, red – human. Horizontal arrows represent communications and feedback: blue – inter-component communication and coordination, orange – collaboration on generated data and information, red – progression of certainty between reports and feedback for program priorities. Vertical arrows represent component sequence of activities and production of reports.

To achieve its predictive surveillance goal, the AFHSC-GEIS has brought together a dynamic team of partners into an integrated, multidisciplinary program with the capability to generate and merge data to produce pre-event advisories and alerts on the emergence of disease outbreaks. Partner activities are organized into three primary predictive components: 1) satellite remote sensing and ecologic niche modeling for ecologic and climatic events, trends and characteristics that otherwise influence the potential for disease outbreaks; 2) arthropod-vector surveillance and geo-spatial mapping for characterizing vector presence, abundance, and disease transmission capability; and 3) animal-host surveillance for detecting vector and pathogen exposure events, and animal-to-animal or animal-to-human pathogen transmission. Another predictive surveillance program component, human-disease surveillance, does not serve a predictive function. Rather it provides evaluation feedback for the overall program’s meta-product, predictive information on human disease threats. The predictive surveillance program components are linked to one another by communications and data-processing channels. Program partners use the channels to integrate and analyze surveillance results from the different components for temporal and spatial relevance. This produces progressively targeted information on emerging threats. (Figure 1)

## Methods

### Program management

The AFHSC-GEIS has oversight and ultimate responsibility for the program’s implementation, and therefore, provides its strategic direction based on DoD priorities and Force health needs. The scientific content and scope of program activities are an expression of consensus among subject matter experts working within the predictive surveillance disciplines. In 2009, they were organized into a steering committee that: 1) promotes the generation of quality-controlled, reproducible data from all program participants; 2) cultivates intra- and extra-DoD predictive surveillance-related clinical and laboratory training and operational research; 3) advocates for the use of standardized surveillance and laboratory methodologies, and evaluates new or novel technologies for applicability; and 4) delineates the roles, responsibilities and mutually-supporting relationships among the AFHSC-GEIS predictive surveillance partners.

### Program model

**Figure 2:**
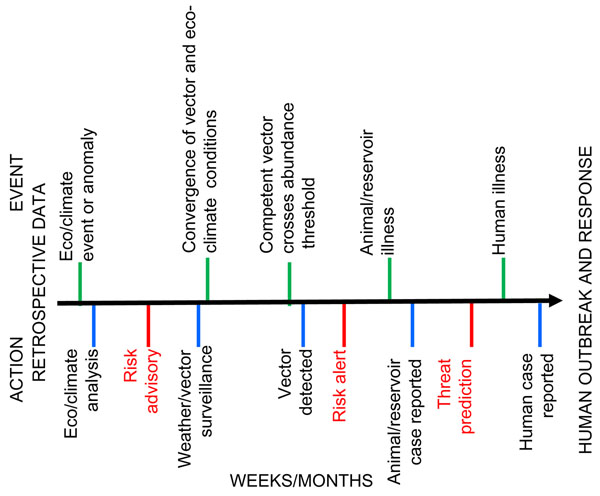
**Predictive Surveillance Model.** Black arrow – timeline for a hypothetical vector borne, zoonotic disease outbreak. The outbreak progresses from left to right. Green bars – natural events in an outbreak’s emergence. Blue bars – the predictive surveillance program activities. Red bars –predictive surveillance program products. The blue and red bars together represent surveillance efforts that detect and follow the progression of the outbreak’s emergence over time. The sequence over time of the green, blue and red bars is set in the model’s configuration, but the spacing between bars varies, according to the outbreak’s pathogen etiology and location.

The core of the program is a model that groups activities into components that conduct surveillance for critical detection points indicative of eco/climatic anomalies, and weather, disease-pathogen, vector, or animal-host events, which are known to be conducive to, and in aggregate, presage the emergence of disease outbreaks in humans. (Figure 2) Program partners generate and analyze derived data from their component activities, then collaborate and coordinate with each other to aggregate and merge their findings into the context of the entire model. The unifying analysis outcome among and between components is disease transmission potential. The selection criteria for applying the model to an etiologic pathogen and geographic location are that they are of stated DOD priority, and that they have direct or indirect temporal and spatial association with environmental trends and events such as El Niño occurrences.[6] Direct associations occur when the expression of disease is dependent on the environment’s direct influence over a pathogen. For example, changes in sea surface temperatures lead to outbreaks of cholera.[7] Indirect associations are most evident for vector-borne diseases, such as Rift Valley fever, where the environment influences the vector’s life cycle, and which in turn influences pathogen presence or transmission. To date, this model has successfully predicted outbreaks of Rift Valley fever in Kenya, Tanzania, Somalia, Sudan, Madagascar and South Africa from 2006 through 2008.[8,9] Host-country ministries of health and agriculture, as well as the World Health Organization (WHO) and the Food and Agriculture Organization (FAO), are currently using the program’s Rift Valley fever advisories and alerts to prepare for and mitigate the impact of Rift Valley fever outbreaks in Africa.

The AFHSC-GEIS program is expanding the use of its model to other priority vector- and water-borne and/or zoonotic diseases including: leishmaniasis, Japanese encephalitis, malaria, chikungunya fever, and hantavirus infections. The extension also covers different ecosystems such as those in southeastern Europe, central Asia, the Middle East, southeast and northeast Asia, and North America [8]. AHFSC-GEIS is consulting with national and international public health authorities to optimize the delivery of the program’s product—comprehensive and reliable information predicting outbreak emergence, through an easily accessible website.

## Model components

### Eco-climate component

The eco-climatic data used in predictive surveillance are derived from two sources: satellite remote sensing for the macro-level influences of climate and ecology, and ground-based measurements (ground-truths) for the micro-level results of weather and habitat conditions. The latter tend to be more precise, but geographically-localized, quantitative measurements. The former are less precise, more qualitative data that give a broad overview of eco-climate events and trends contributing to disease outbreak potential. Both are used in tandem for meaningful analysis.

#### Remote sensing

**Figure 3:**
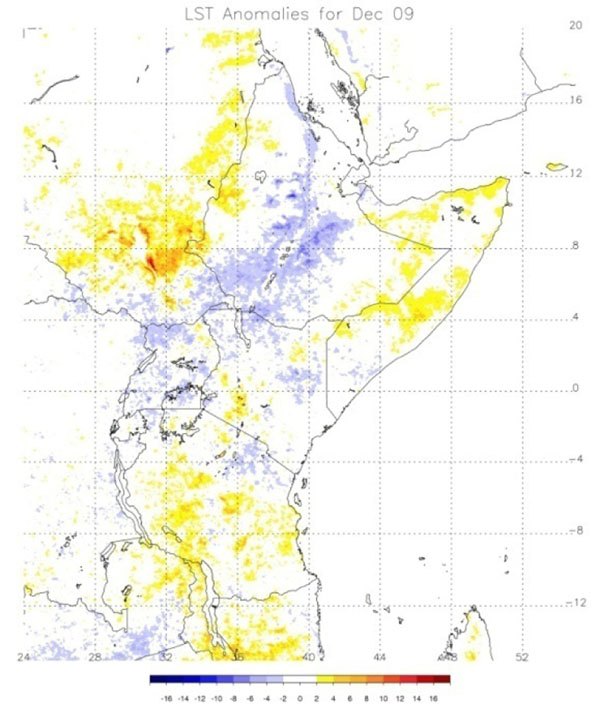
**Land Surface Temperature (LST) Anomaly for Eastern Africa, December 2009.** Blue – cooler than average land surface temperatures. Yellow/orange/red – warmer than average LST.

**Figure 4:**
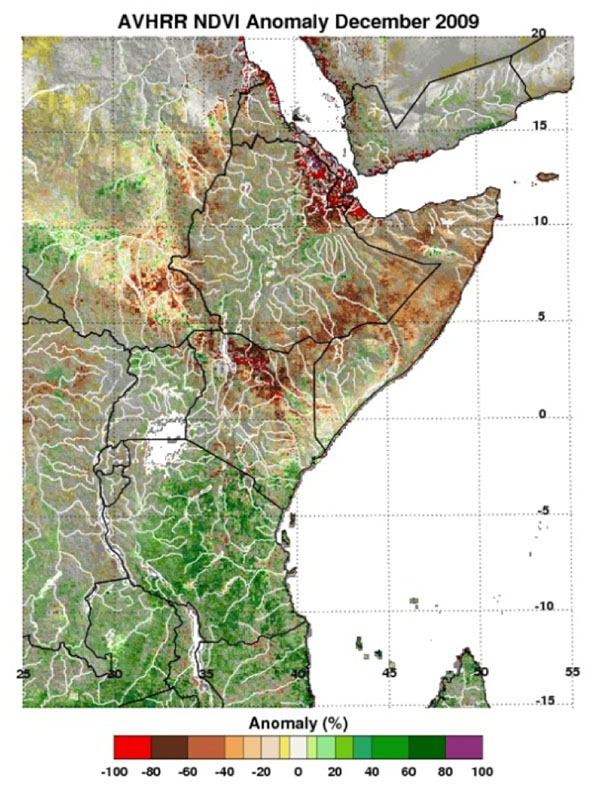
**AVHRR-N17 NDVI Anomaly for Eastern Africa, December 2009.** Green – greater vegetation growth as a surrogate for sufficient and prolonged rainfall. Brown – less vegetation growth signaling less than normal rainfall.

**Figure 5:**
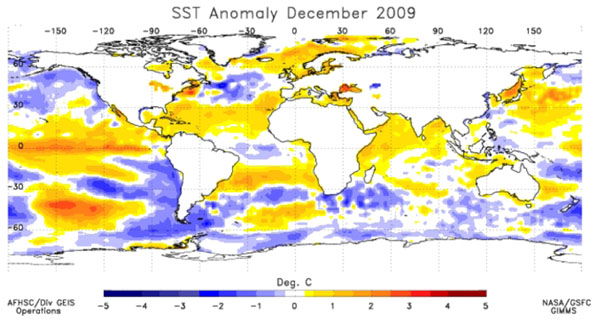
**Global Sea Surface Temperature (SST) Anomaly for December 2009.** Yellow and orange – warmer than normal SST in the equatorial Pacific and Indian Oceans. This is characteristic of a warm ENSO (El Niño) event.

**Figure 6:**
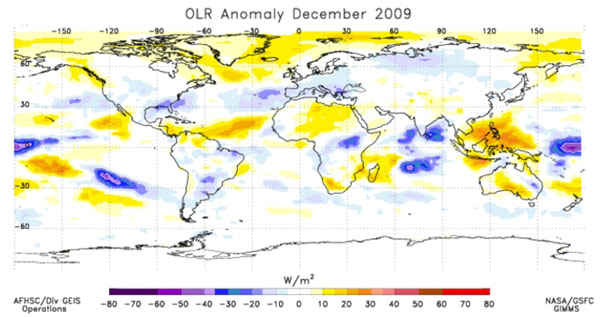
**Global Outgoing Longwave Radiation (OLR) Anomaly for December 2009.** Blue areas - negative OLR anomalies in the eastern Pacific and Indian oceans and in east central Africa are indicative of higher than normal rainfall in these areas. Yellow to orange – lower than normal rainfall.

**Figure 7:**
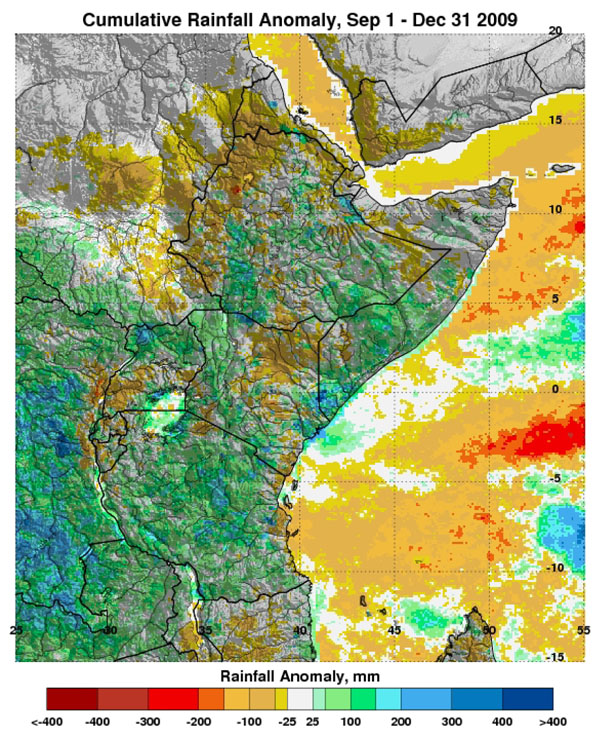
**Cumulative Rainfall Anomaly for Eastern Africa, Sept. 1 to Dec. 31, 2009.** Blue and green – Rainfall totals of between 100 and 300 mm above normal being detected in nearly all of the region except in north and central Kenya where November rains were light.

**Figure 8:**
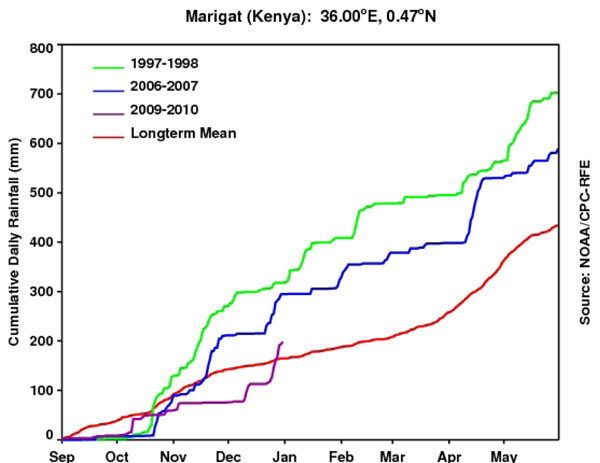
**Cumulative Rainfall Totals for Marigat, Kenya.** The two most recent El Niño seasons are represented in green – 1997-1998, and blue – 2006-2007. Red – the long-term rainfall mean. Purple – rainfall amount during the 2009-2010 season through Dec. 31, 2009. Not shown – heavy rainfall occurred during the last week of December 2009 at this site and others throughout the central Rift Valley.

The Global Inventory Modeling and Mapping System (GIMMS) group at the National Aeronautics and Space Administration’s (NASA) Goddard Space Flight Center monitors global-scale indicators of inter-annual climate variability, for example the El Niño Southern Oscillation (ENSO). The group performs detailed analyses of satellite-generated datasets on land surface temperature (LST), [10] normalized difference vegetation index (NDVI) [11,12], sea surface temperature (SST), [13] outgoing long-wave radiation (OLR) [14] and rainfall [15]. The group uses time series analyses for assessing macro-level ecologic dynamics that signal the persistence of an eco-climate trend with known association to disease outbreak emergence (Figures 3, 4, 5, 6). GIMMS then produces illustrative maps and graphics that form a risk assessment, the first product of our model’s prediction process. For example, in the Rift Valley fever project, GIMMS uses the concurrence of warmer SSTs in the central and eastern Pacific (>1^o^ C), and in the western equatorial Indian Ocean (>5^o^ C), as its analysis’ initial critical detection point. Historical observations and expert analyses link these SSTs to excessive rainfall over Eastern Africa. When the resulting greener-than-normal conditions (vegetation growth) continue for longer than a three-month period, an ecology emerges for mosquito-presence and survival [1]. However, because the remote sensing datasets are snapshots of large geographic areas, and therefore are only surrogates for actual weather-related conditions, they must be augmented with quality-controlled direct measurements, i.e., environmental temperature or rainfall volume at representative localities on the ground (Figures 7, 8).

**Figure 9:**
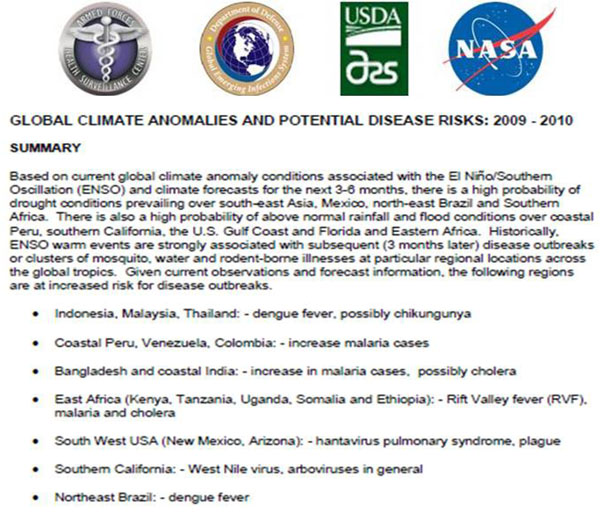
**The AFHSC-GEIS and NASA Predictive Surveillance Advisory, August 2009**. Predictive surveillance advisories are sent to AFHSC-GEIS Predictive Surveillance program partners, DoD public health authorities, and other national and international organizations. The advisories also are publicly shared on an open-access website (ftp://rvf:geis@pengimms.gsfc.nasa.gov) when eco-climatic events and trends suggest that disease outbreaks may arise in the coming several months. The August 2009 advisory was not geographically limited because the expected El Niño conditions were expected to have global impact.

GIMMS’ analyses result in advisories based on detections of early shifts in eco-climate trends. As these trends start to produce indicators of rising disease risk in specific geographic locations, the advisories transition to more certain alerts of threat emergence. GIMMS disseminates advisories and alerts to Federal collaborating agencies (DoD overseas laboratories and public health and medical research agencies [16], U.S. Agency for International Development, Centers for Disease Control and Prevention, and U.S. Department of Agriculture), and international partners such as WHO and FAO. When used in conjunction with ground-truth information, remote sensing targets subsequent surveillance activities to those locations most at risk. Such targeting permits intensive vector- and animal-host surveillance for pathogen transmission and disease emergence where it is most needed [17]. In August 2009, the GIMMS group issued an early warning advisory on an emerging global disease risk from autumn 2009 through spring 2010 (Figure 9).

#### Ecologic niche modeling

Remote sensing products and climatic averages such as rainfall and ambient temperature are used in ecologic niche modeling (ENM). ENM characterizes those habitat and geographic conditions suitable for maintaining disease vectors and animal hosts, and thus those areas suitable for the transmission of disease pathogens during outbreaks. Estimations of ecologic niches help investigators map precisely where a vector or host may live and thrive, providing vital information for predicting where a vector-borne or zoonotic disease may occur [18-20]. Examples of diseases targeted by the AFHSC-GEIS program’s ENM include JE and malaria in the Republic of Korea (ROK) [21-24].

**Figure 10:**
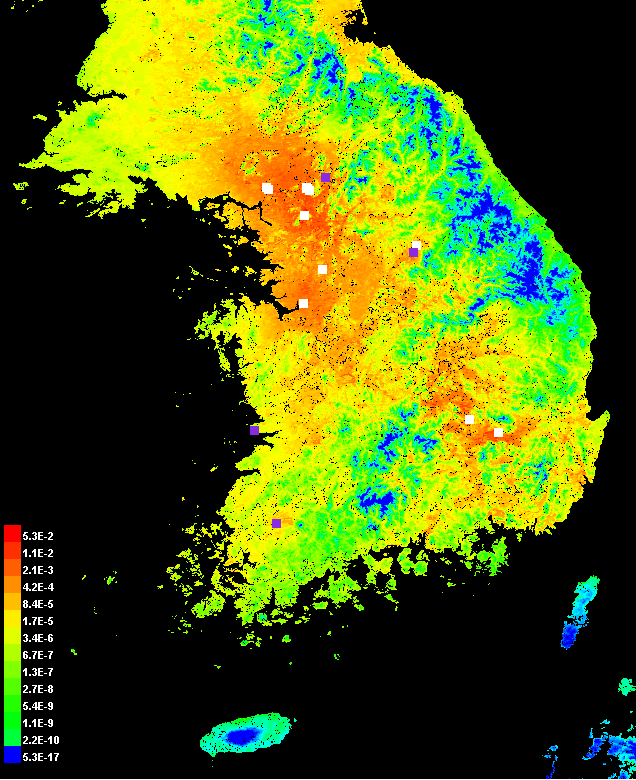
**Predicted Distribution of *Cx tritaeniorhychus* in the Republic of Korea**. Red and orange – higher probability of *Cx tritaeniorhychus* occurrence. Blue – lower probability of *Cx tritaeniorhychus* occurrence (corresponds to higher elevations). Purple and white dots – mosquito collection sites. White dots were used for model building (training points) while purple dots were withheld from the model and used for testing the accuracy of the model. This work demonstrates that cropland conditions, primarily rice fields and minimum temperature in the summer, are positive factors in predicting this mosquito’s distribution; whereas forested hills and mountains >1000 m elevation, are negative factors.

For JE, the Uniformed Services University of the Health Sciences (USUHS) partners use the maximum-entropy approach, Maxent [25], to model the occurrence of the primary JE vector *Culex tritaeniorhynchus*[19]. This approach uses environmental variables (e.g., average minimum monthly temperature, total monthly precipitation, vegetation indexes, land cover and elevation) and geo-referenced mosquito collections to derive a vector’s spatial range. USUHS partners then determine the controlling environmental factors for *Cx. tritaeniorhynchus* abundance, distribution and periodicity. The intended result is the identification of a putative detection point for predicting JE risk to U.S. Forces in the ROK (Figure 10). In the future, USUHS partners will test this ENM application to JE in other countries such as Thailand.

**Figure 11:**
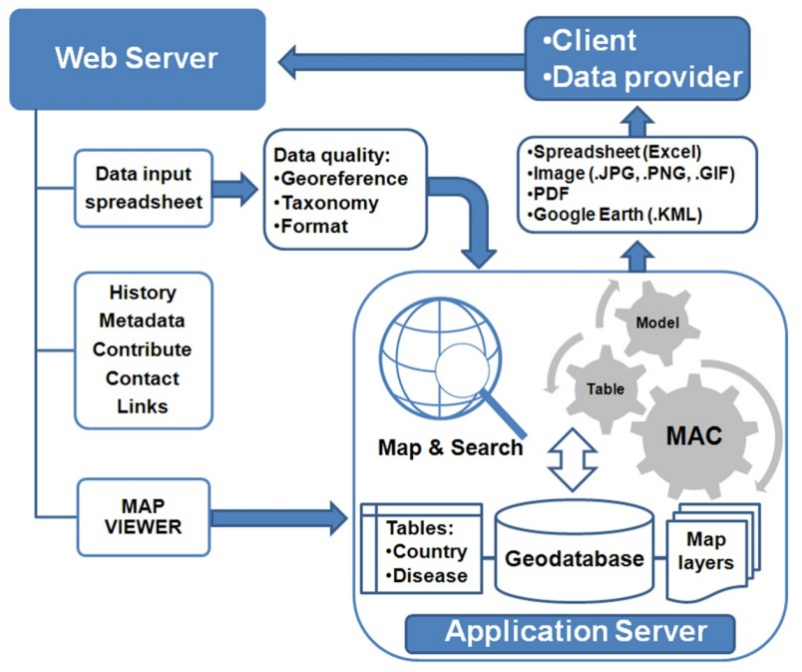
**Overview of MosquitoMap.** A client or prospective data provider accesses the web server hosting MosquitoMap. A downloadable spreadsheet is available with instructions regarding data requirements. Data can be submitted via email. A link is also available to the map viewer hosted on an application server running ArcGIS Server 9.3. The client can map and search the geodata-base, or use the Mal-area calculator (MAC) to quantify the overlap of models of vector, disease and human distribution. Output is available to the client in various formats. Figure reproduced from Foley et al. (2010)

MosquitoMap (http://www.mosquitomap.org) is an AFHSC-GEIS online product that incorporates ENM as a predictive surveillance tool for vector surveillance. At its core is a data-curation process that organizes and standardizes accurate mosquito collection, distribution data, and pathogen-transmission models [26-28]. Using MosquitoMap, its developers at the Walter Reed Biosystematics Unit (WRBU), Walter Reed Army Institute of Research (WRAIR) make accurate, high-resolution distribution maps of where vectors are predicted to exist. MosquitoMap is paired with the Mal-area Calculator (MAC), a raster overlay analytic tool that maps and quantifies the extent of where vectors, disease pathogens, and humans co-occur (Figure 11).

During 2009, WRAIR-WRBU partners focused on validating MosquitoMap’s vector distribution models for the ROK [29]. WRAIR-WRBU also began making vector map-layers for Southeast Asia to expand the geographic functionality of MosquitoMap. During 2010, partners will incorporate MosquitoMap into a new application—VectorMap, which will include sand fly and tick data and models (Foley DH, personal communication).

### Vector component

The second program component, the vector component, is currently organized to support the niche modeling applications described above, and promote the identification of critical detection points for other vector-borne diseases included in the AFHSC-GEIS predictive surveillance program. While the program’s vector surveillance activities historically centered on mosquitoes (focus: Rift Valley fever, JE, malaria), in 2009 they expanded to include sand fly (focus: leishmaniasis) and tick (focus: rickettsioses) surveillance.

#### Mosquito

To improve Maxent accuracy and broaden its usage, investigators at the Armed Forces Research Institute of Medical Sciences (AFRIMS) adapted an RT-PCR assay for detecting JE and other flaviviruses in mosquito specimens. This improved diagnostic capability is used to increase the validity of the vector component’s identified critical detection point for JE and other flaviviruses. Also, USUHS investigators are adding a vegetation height evaluation protocol to enhance Maxent’s ecologic specificity. Both these efforts are tailored toward using the Maxent application on flavivirus threats in Southeast Asia.

**Figure 12:**
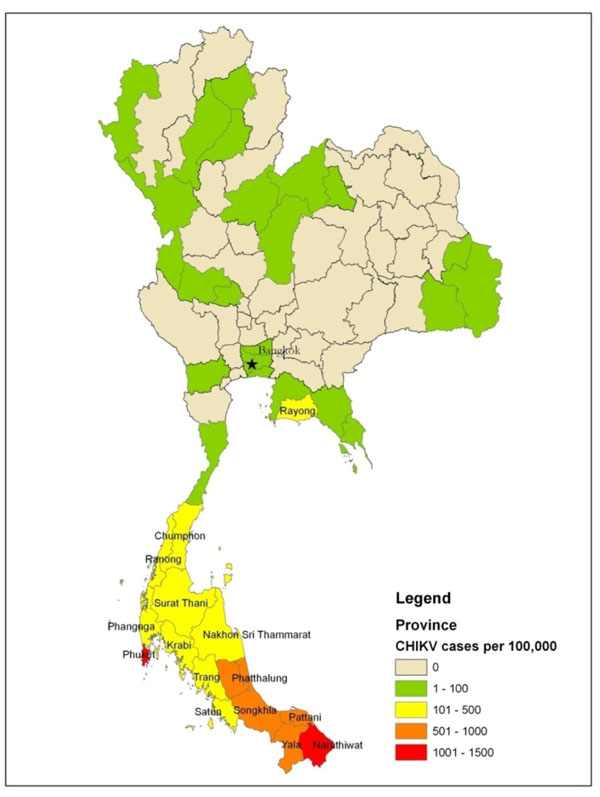
**Distribution of 2009 CHIKV Cases Showing a Clear Concentration of Cases in the Southern Peninsula**. Data reflects suspected cases (per 100,000 province inhabitants) reported by the Bureau of Epidemiology, Ministry of Public Health, Thailand. Data available at http://203.157.15.4/chikun/chikun/situationy52/chikun_20091231520.pdf (in Thai).

**Figure 13:**
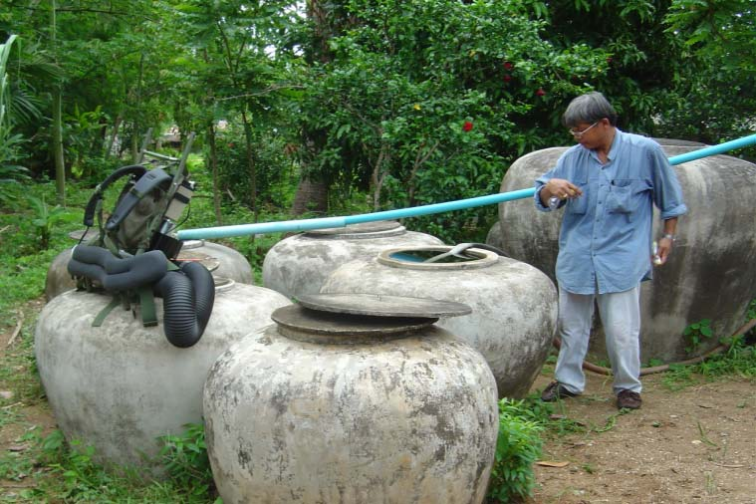
**Inspection of Water Cisterns in Southern Thailand as Part of a Chikungunya Virus Outbreak Investigation**. AFRIMS personnel are collecting mosquitoes throughout Thailand as part of a niche modeling effort of CHIKV vector distributions. Vector density and distribution are the likely key drivers of the restricted distribution of CHIKV cases observed in Thailand in 2009.

In anticipation, AFRIMS hosted a workshop in 2009 on ENM for 21 scientists from Thailand and the Philippines. The pathogen focus for this workshop was chikungunya virus (CHIKV), the cause of a severe, ongoing outbreak (>48,000 reported cases in 2009) in southern Thailand.[30] (Figure 12) AFRIMS engagement with the Thai Ministry of Public Health (MoPH) in CHIKV field investigations reinforces AFRIMS’ reputation as a valued regional partner in mosquito control, risk assessment, and determiner of the driving forces behind this outbreak (e.g., factors in virus transmission, vector competence, and landscape epidemiology). (Figure 13) AFRIMS virologists responded to the outbreak by conducting real-time testing of both human blood and mosquito samples for virus presence and virulence (A226V mutation).[31] Characterization of circulating viruses is possible because AFRIMS has biosafety level (BSL)-3 capability to culture CHIKV. All outbreak information generated by AFRIMS is shared with both the Thai MoPH and Royal Thai Army for their public health decision-making. In total, these efforts not only contribute to the resolution of this outbreak, they will inform future inclusion of CHIKV in the AFHSC-GEIS predictive surveillance program.

#### Sand fly

During 2009, USUHS trained a WRAIR entomologist to apply ENM to sand fly surveillance. This represents a first step by the predictive surveillance program to cover non-mosquito vectors of disease. In conjunction with this training, initial investigations were done to test the application of remote sensing to sand fly larval-habitat characterizations, and their use in determining critical detection points for leishmaniasis transmission in the Middle East [32].

**Figure 14:**
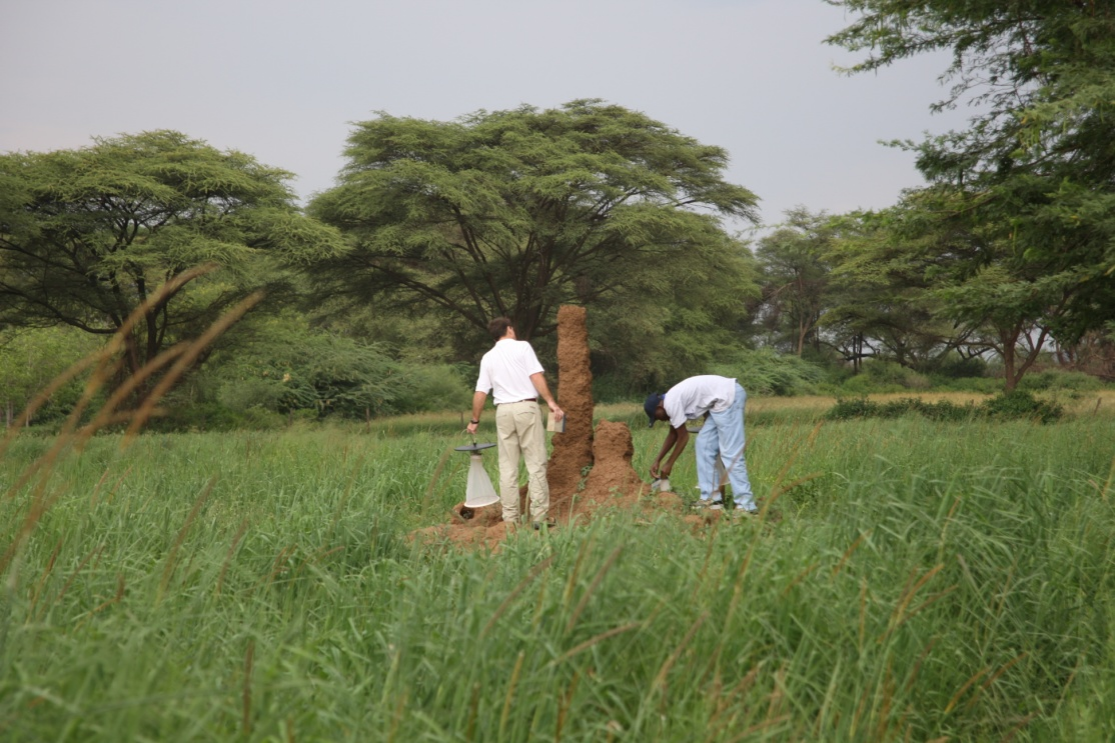
**Sand Fly Surveillance in Kenya.** Traps are set on the base of a termite mound in Marigat, Baringo district, Kenya. Termite mounds are one of the natural habitats for *Phlebotomus martini*, a vector of visceral leishmania in East Africa.

At the same time, AFHSC-GEIS partners at the U.S. Army Medical Research Unit-Kenya (USAMRU-K) and the Kenya Medical Research Institute (KEMRI) conducted large-scale surveillance for sand fly presence at over 200 sites in three distinct geographic regions of Kenya. Approximately 3,500 sand flies were identified and tested for *Leishmania* spp. infection using polymerase chain reaction (PCR) techniques. This traditional surveillance work forms the basis for future ENM and predictive surveillance for leishmaniasis in that country and potentially elsewhere in sub-Saharan Africa (Figure 14).

To date, there have been two major findings with epidemiological and predictive surveillance implications in Kenya. First, *Leishmania major*-infected sand flies (species undetermined) were detected in two regions not previously known for leishmaniasis transmission: Isiolo and Lamu (Figure 11). Second, *Phlebotomus orientalis*, a known vector of visceral leishmaniasis (VL) in Sudan, was detected in large numbers in Isiolo and at another site, Garissa (Musila LA, personal communication). Garissa is considered a non-endemic area for cutaneous leishmaniasis (CL) and VL [33-36]. *P. orientalis* is rarely collected in Kenya as a whole [37].

Interestingly, USAMRU-K investigators are receiving reports from the Wajir area in northeast Kenya on possible VL cases among inhabitants and refugees from Ethiopia and Somalia. This province is not traditionally considered endemic for leishmaniasis either. However, the area did undergo drought conditions after the 2006-2007 El Niño rains; and similar reports of leishmaniasis were made during the aftermath of the El Niño events of 1997 and 2000-2001 (and possibly historically since the 1930s) [38]. Therefore, USAMRU-K and KEMRI researchers are investigating this apparent environmental association. If a temporal relationship exists between El Niño events, sand fly activity, and leishmaniasis occurrences, it will provide a solid foundation for expanding predictive surveillance capability in Kenya beyond the Rift Valley fever project.

#### Tick

Tick surveillance is also conducted in the predictive surveillance program’s vector component. As an initiation point, the program is using rickettsiae as the pathogen focus. During 2009 at Fort Eustis, Virginia, partners from the Naval Medical Research Center (NMRC) collected three species of ticks known to parasitize humans (*Amblyomma americanum*, *Dermacentor variabilis*, *Amblyomma maculatum*). The ticks were tested for rickettsiae (*Borrelia lonestari* and *Ehrlichia chaffeensis*), using quantitative real-time PCR (qPCR) assays and multilocus sequence typing (MLST).

Researchers tested 108 *A. americanum* ticks pooled in 33 samples, and found that 33 (100 percent) of the pools had positive reactions to *Rickettsia amblyommii* (unknown pathogenicity), two (6.1 percent) for *B. lonestari* (unknown pathogenicity), and one (3 percent) for *E. chaffeensis* (human monocytic ehrlichiosis). All six pools (14 individual ticks) of *D. variabilis* were negative for evidence of rickettsiae. *Rickettsia parkeri*, a newly recognized pathogen, [39] was found in one *A. maculatum* tick. This is significant because until 2002, all confirmed cases of tick-borne spotted fever in North America were attributed to only one pathogen, *Rickettsia rickettsii*, the cause of Rocky Mountain spotted fever. Previously, tick-borne rickettsiae other than *R. rickettsii* were considered non-pathogenic in the United States. Between 2002 and 2006, *R. parkeri* infection was detected in six cases (two DoD beneficiaries) in the southeast United States. This detection and subsequent investigations of *R. parkeri* infection in the U.S. are being used to build future predictive surveillance capability on this and other tick-associated diseases.

Overseas, an astute AFHSC-GEIS partner in the ROK noted that in 2004 two service members presented at the 121^st^ Hospital with eschars typical for spotted fever group (SFG) and scrub typhus group (STG) rickettsial infections. This observation prompted a serology study in 2005 which resulted in the first evidence that SFG rickettsial infections are associated with deployments of U.S. military personnel to Korea, 181 seropositive of 8,918 tested (2 percent).

### Animal component

While it is generally accepted that eco-climate trends and events can influence disease transmission from animals to humans [40,41], given the complexity of animal population dynamics, bionomics and behaviors, considerable research is needed to identify the determinants of that association. As a starting point, the AFHSC-GEIS partners working in this component are focusing on rodent-borne hantavirus diseases.

**Figure 15:**
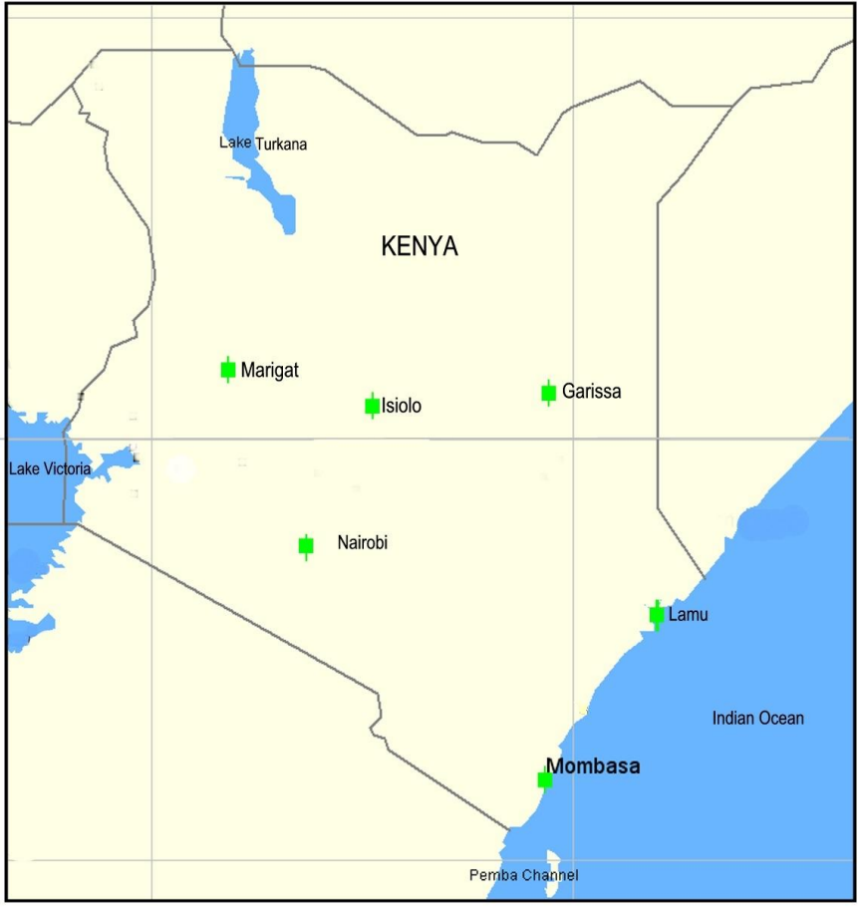
**Map of Kenya Showing Surveillance Sites: Arthropod Vectors and Animal Hosts. ***Lieshmania major*, a protozoan parasite causing zoonotic cutaneous leishmaniasis, has been detected in Isiolo in the central part of Kenya and Lamu along the northern coast. The visceral leishmaniasis vectors, *Phlebotomus orientalis*, and *P. martini* were detected in Garissa in Kenya’s Northeast Province and in the Rift Valley village of Marigat respectively. Hantaviruses have been found in both Marigat and Garissa.

In Kenya, USAMRU-K discovered hantaviruses in live rodents from Marigat and Garissa (Figure 15). These viruses are now being characterized at the U.S. Department of Agriculture, Fort Collins, Colorado, and their presence in Kenya will be analyzed for correlation with the already existing eco-climate data from the Rift Valley fever project. This cross-analysis of datasets will allow investigators to explore how the eco-climate conditions and trends interface with hantavirus infections in East Africa. It will also facilitate a Naval Medical Research Unit No.3, Cairo, Egypt (NAMRU-3) and NASA analysis of hantavirus outbreaks in the Ukraine. Interestingly, NASA partners observed some geologic similarities between Kenya and the Ukraine. If validated, this observation will be useful in determining the predictive surveillance model’s indicators of critical detection points for the emergence of hantavirus outbreaks.

In the ROK, U.S. and Korean partners are conducting small mammal surveillance to elucidate hantavirus exposure-risk at U.S. field training sites. Recently, these investigators discovered three new hantaviruses (Soochong, Muju, and Imjin viruses) [42-44].

To complement the vector component work on rickettsial infections, Naval Medical Research Unit No. 2, Jakarta, Indonesia (NAMRU-2) has found rickettsiae in small peri-domestic mammals on the islands of Java, Kalimantan, Sulawesi and Sumatra in Indonesia. In a total of 491 animals (including shrews, mice and eight species of rats), 26 percent were seropositive for SFG rickettsiae, 14 percent for typhus group, and another 10 percent for STG rickettsiae. Interestingly, *R. felis* and another SFG Rickettsia spp. were found associated with 208 fleas (*Xenopsylla cheopis*) from these rodents. Similar studies were conducted with partners in the ROK [45] and in Peru (37 of 742 {5 percent {ectoparasites from domestic animals were positive for rickettsiae, including *R. parkeri* {Tidewater spotted fever}, *Candidatus*, *Rickettsia andeanae* {unknown pathogenicity}, and *R. felis* {*flea-borne spotted fever*}).

### Human component

As the AFHSC-GEIS predictive surveillance model is framed, the human component comprises two aspects of human-based surveillance and analysis. The first is human disease and case detections from laboratory diagnosis or syndromic surveillance. This human disease surveillance is described elsewhere [46] and will not be discussed here, except to say that it helps the predictive surveillance program focus on its ultimate subject, human disease. It provides the retrospective and prospective epidemiologic information necessary to target, analyze and refine the program’s model.

The second aspect, and the one that is integral to the predictive surveillance model itself, is the identification and characterization of those specific human factors that can serve as critical detection points for human-disease outbreak emergence. As an initial pilot study (using a directly-transmitted disease - cholera), the Johns Hopkins University Applied Physics Laboratory (JHU/APL) assessed the demographic, economic, environmental, and climatic variables associated with outbreaks in Africa during 1995-2005. The study’s putative contributing factors for outbreaks included qualitative and quantitative sanitation and water source contamination, rainfall, and water management (i.e., flooding) factors, human housing density and quality (i.e., peri-urban sprawl), and human migration and living-practices associated with displaced persons or refugees. This study showed good correlation between these factors and cholera outbreaks. It also showed that the occurrence of a cholera outbreak in a neighboring province during the previous month could also be a predictive factor [47]. This study sets the stage for the analysis and definition of eco-climate and pathogen transmission factors involved in human disease outbreak emergence.

### Cross-component activities

#### Diagnostic support

As each of the above components strives to identify the critical detection point(s) which signal(s) a potential for pathogen transmission, it is understood that the predictive surveillance model’s underpinning is pathogen presence or absence in a geographic area, not the intrinsic behavior of the pathogen itself. This necessitates the inclusion of accurate diagnostic testing capability in the program’s activities. During 2009, this aspect of the program was best exemplified by work done by NMRC on rickettsiae detection and characterization.

After developing and producing rickettsiae diagnostic assays, NMRC used them to test arthropod vectors, animal hosts and specimens from human cases for rickettsial infections [48]. NMRC has tested for and confirmed the presence of *Rickettsia felis* (flea-borne spotted fever) in febrile patients from northeastern Kenya. By developing and providing an Enzyme-linked Immunosorbent Assay (ELISA) test specific for SFG rickettsiae antibodies against *Rickettsia conorii*, NAMRU-2 confirmed the presence of SFG rickettsioses among fever patients in Cambodia (Kasper M, personal communication).

NAMRU-2 has also performed SFG rickettsiae-specific ELISA testing on 368 sera collected from residents of Jakarta, Indonesia. In this work, NAMRU-2 found SFG rickettsiae-specific antibodies in 60 serum samples (16.3 percent), and of these, five paired samples showed a four-fold rise in titer indicating acute SFG rickettsiosis. Likewise, NAMRU-2 was able to test pooled ectoparasites from rodents collected in Jakarta, and found 2/40 (5 percent) positive for SFG rickettsial DNA.[49]

Through the predictive surveillance program, NMRC will continue to provide DoD investigators with technical assistance and assays for detecting rickettsial diseases (including Rocky Mountain spotted fever, Tidewater spotted fever, murine typhus, flea-borne spotted fever and other rickettsioses) that present emerging and re-emerging risk to DoD military, civilian, and family members throughout the world.

#### Dataset standardization

To ensure reliability and validity, the AFHSC-GEIS program is promoting consensus on and the establishment of dataset standards for the program’s component activities. Dataset standardization is necessary for conducting meta-analyses on the results of program activities, and merging them into meaningful information for advisories, alerts and predictions. For example, precise taxonomic and geo-referenced collection data are essential for understanding vector and host bio-geography, ecology and distributions, and the impact of environmental changes on them. The data are essential for determining pathogen transmission factors associated with outbreaks. Accurately geo-referenced vector collection data must be matched spatially and temporally with remote sensing data of an appropriate resolution to answer questions about the environmental determinants of vector-borne disease distribution. While the methodology of collecting datasets may vary according to the instruments used, the end results, the language and content of the datasets must be concordant in order to facilitate a seamless progression of surveillance results between AFHSC-GEIS partners and program components. Partners at WRAIR-WRBU have published recommendations on mosquito collection data standards, and these are being considered as the standard operating procedure (SOP) for mosquito collections sponsored by AFHSC-GEIS [50]. Similar SOPs will be developed for sand fly and tick collections.

## Conclusions

Looking to the future, the AFHSC-GEIS predictive surveillance program in its entirety, its management framework of the AFHSC-GEIS Predictive Surveillance Steering Committee, and its scientific model that provides direction to program surveillance and analysis activities, will continue to support the detection and reporting of emerging vector- and water-borne and zoonotic disease outbreaks of importance to DoD.

Under AFHSC-GEIS leadership, the program will refine and expand its model of disease outbreak emergence surveillance. It will build and elaborate upon those component activities that optimize collaborations between partners. It will continue to define those critical detection points that represent the model’s signal that an outbreak is emerging. The program will continue to advocate for focused, pre-emptive public health action to prevent or mitigate human morbidity and mortality, as well as the destabilizing effects of infectious disease outbreaks.

In the near term, the predictive surveillance program is expanding upon its initial Rift Valley fever success. It will continue to characterize and validate identified eco-climate anomalies for predictive surveillance application under different ecosystem and habitat conditions. Partners will continue to identify disease-associated vectors and hosts with demonstrated periodic or cyclic disease-transmission pathways that are plausibly associated with eco-climatic events and trends. Partners will investigate vector- and water-borne and zoonotic diseases for which the program’s model might be applicable, and most importantly, which present operational risks to DoD force. This expansion is now underway with the program’s activities on JE, leishmaniasis, hantavirus and chikungunya fever. The program is initiating pilot activities on Crimea-Congo hemorrhagic fever, Ebola and other viral hemorrhagic fever outbreaks, and malaria.

By continuing efforts in strengthening pathogen detection capabilities, program components are improving the sensitivity and specificity of the model’s critical detection points. As the program expands, partners will learn more about the model’s capabilities and determine where, for what and under what conditions the model does or does not apply. This will necessitate an incremental growth for the program, subjecting model usage to continuous quality assurance and applicability assessments. As the program matures, AFHSC-GEIS will facilitate the appropriate transfer of the components’ operations to those partner organizations best suited to ensure sustainable, quality-assured predictive surveillance.

The success of predictive surveillance will depend on the recognition that it is founded on a meta-system of different surveillance activities, linked by communications, coordination and collaboration between multiple, diverse disciplines, and producing advisories and alerts, which have progressively more certainty over the course of an outbreak’s emergence. No single component can provide sufficient information for accurate predictions. Just because we detect an El Niño event, does not mean that a disease outbreak will occur. Similarly, if a vector is present in a locality, it does not mean that the pathogen is present or that disease will be transmitted to humans. Human activities may permit, exacerbate, or prevent human exposure to the risk of disease transmission. It is only when all relevant components of the predictive surveillance model are applied diligently and with scientific rigor to the synthesis of a complete agent-vector-host-environmental scenario, that the program will produce reliable advisories, alerts and predictions.

The role of AFHSC-GEIS in the predictive surveillance program as a whole is to provide an agile, investigative platform for continuously testing different scenarios, combinations of geographic areas, vector- and host-species characteristics, and pathogens to facilitate the development of a stable of predictive surveillance models for an array of different infectious disease outbreak scenarios. In the end, however, even once given, a prediction, advisory, or alert cannot prevent outbreaks. It can only inform the decision-making process for timely, effective public health action.

## Competing interests

The authors declare no competing interests.
